# Starch-Based Super Water Absorbent: A Promising and Sustainable Way to Increase Survival Rate of Trees Planted in Arid Areas

**DOI:** 10.3390/polym13081314

**Published:** 2021-04-16

**Authors:** Pattra Lertsarawut, Thitirat Rattanawongwiboon, Theeranan Tangthong, Sakchai Laksee, Tanagorn Kwamman, Butri Phuttharak, Phayao Romruensukharom, Phiriyatorn Suwanmala, Kasinee Hemvichian

**Affiliations:** 1 Thailand Institute of Nuclear Technology (Public Organization), Nakhon Nayok 26120, Thailand; pattra@tint.or.th (P.L.); thitirat@tint.or.th (T.R.); t_ranan@hotmail.com (T.T.); sakchai@tint.or.th (S.L.); tanagorn@tint.or.th (T.K.); piriyatorn@tint.or.th (P.S.); 2 Chachoengsao Rubber Regulatory Center, Chachoengsao 24160, Thailand; bp_shan@hotmail.com (B.P.); phayaor@hotmail.com (P.R.)

**Keywords:** cassava starch, super absorbent polymer, super water absorbent, radiation-induced graft polymerization, field test, rubber tree, drought, SAP, SWA, acrylic acid

## Abstract

This research aimed to scale up the production of starch-based super water absorbent (SWA) and to validate the practical benefits of SWA for agricultural applications. SWA was successfully prepared in an up-scaling production by radiation-induced graft polymerization of acrylic acid onto cassava starch. Chemical characterization by FTIR and thermal characterization by TGA showed results that differentiated starting materials from the prepared SWA, thus confirming effective preparation of starch-based SWA via radiation-induced graft polymerization. SEM results visibly revealed a highly porous morphology of the synthesized SWA, substantiating its high swelling ability. Results from the field tests, performed for two seasons, revealed that the prepared SWA was able to increase the survival rate of young rubber trees planted in arid area by up to 40%, while simultaneously enhancing the growth characteristics of the young rubber trees.

## 1. Introduction

Thailand is recognized as one of the world’s leading exporters of a number of agricultural products, such as coconut, sugarcane, rice and rubber. Rubber plantations are very common in the southern region of Thailand, where there is an abundance of rain almost throughout the year. In recent years, farmers in the northeastern part of Thailand have been increasingly growing rubber trees, due to a number of factors, especially price. However, the rainfall in the northeastern parts is highly irregular, resulting in arid conditions and ultimately low survival rate (20–40%) of rubber trees. One of the most promising solutions to this problem is the application of super absorbent polymer as a local reservoir to release water and maintain moisture balance.

Super water absorbent (SWA) is an extremely hydrophilic polymer that is crosslinked into a three-dimensional (3D) network and has a unique ability to absorb a considerable amount of water (at least a few hundred times its original dry weight). The synthesis and characterization of SWA have been studied and reported [1,2,3,4,5,6,7,8,9,10]. SWA can be prepared via free-radical initiation using chemical initiators [11,12,13] or via radiation technology using gamma or electron beam irradiation [14,15,16,17,18,19,20,21,22,23,24,25,26,27]. During the past few decades, radiation processing has become one of the most convenient technologies utilized to improve properties of various polymers, both natural and synthetic [28,29,30,31,32,33]. Cost-effective and efficient, this powerful technology can induce crosslinking, degradation and/or grafting without any initiators, at ambient temperature, while concomitantly offering convenient processing in diverse sizes and shapes.

During the past decade, the preparation of SWA from natural polymers, especially polysaccharides such as starch and cellulose, has been increasing [34,35,36,37,38,39,40,41,42,43,44,45,46,47,48,49]. This is predominantly due to the fact that natural polymers are environmentally friendly. In many countries, these natural polymers are abundant and cheap, thus providing a highly cost-effective substitute for non-biodegradable petroleum-based polymers. In Thailand, cassava starch (CS) is abundant and economical. In addition to being inexpensive, CS is also biodegradable, compostable, non-toxic and renewable, thus making it a promising natural polymer in a bio-circular-green (BCG) economy. The development of starch-based SWA can bring about not only the value addition of cassava starch, but also the declined import of synthetic polymer-based super absorbent polymers. Our previous work [50] has demonstrated that starch-based SWA can be successfully prepared from radiation-induced graft polymerization of acrylic acid (AA) onto CS in a laboratory scale. The preliminary results from this work have also revealed that the presence of SWA has considerable effects on seed germination of corn seeds and growth of young corn plants, implying a potential for scaling up of SWA for agricultural applications.

A number of research works on the agricultural applications of SWA [51,52,53,54,55] as well as the effects of SWA on soil and/or water [56,57,58,59] have been reported. Nonetheless, field tests of SWA in a sizable scale with a significant sample population, has hardly been investigated. Consequently, the two major objectives of this research are (1) to verify the possibility of up-scaling production of starch-based SWA and (2) to subsequently validate the practical benefits of the prepared SWA for agricultural applications by applying SWA in a real agricultural field. In this research, the field tests of starch-based SWA were carried out, for two separate seasons, with young rubber trees. The field tests with young rubber trees were carried out in an arid area, for two seasons for 6–7 months without watering, to essentially emphasize on investigating the effects of SWA on the survival rate and growth characteristics of the young rubber trees.

## 2. Materials and Methods

### 2.1. Materials

Cassava starch (12.8% moisture) was procured from Siam Quality Starch Co. Ltd., Pathum Thani, Thailand. Potassium hydroxide (KOH, 95%) was obtained from UNID, Inchon (South Korea). Acrylic acid (AA) (purity ≥ 99.5%) was purchased from LG Chem Ltd. Jeollanam-do (South Korea). Polyacrylic acid (PAA) (MW = 750,000 g/mol) was supplied by Sigma-Aldrich, St. Louis, MO (USA). All chemicals used are industrial grade and were utilized without further purification.

### 2.2. Up-Scaling Production of Starch-g-PAA (SWA)

**Scheme 1:**
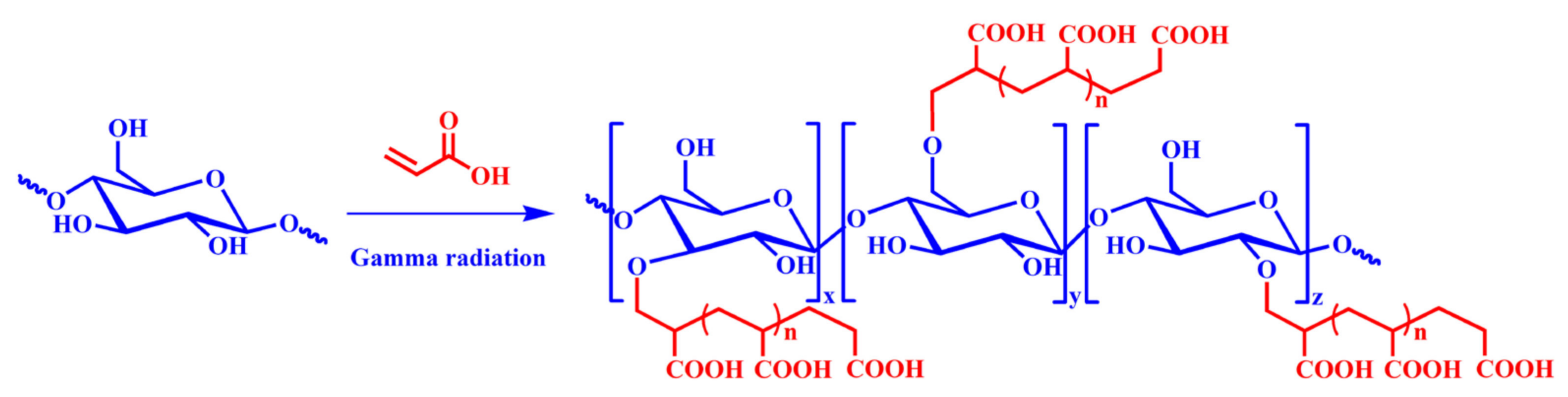
Degree of grafting and swelling ratio.

Scheme 1 illustrates the synthesis of SWA from radiation-induced graft polymerization of AA onto CS. The preparation of SWA in laboratory scale was described in details in our previous work [50]. The following method was used to prepare SWA in an up-scaling production for this study. In a 150 L stainless steel reactor, 7.5 kg of cassava starch was mixed with 105 L of filtered water. The mixture was mechanically stirred for 30 min. 6 kg of KOH was dissolved in 7.5 L of filtered water in a water-cooled reactor. KOH solution was left to cool down to room temperature, after which the solution was gradually mixed with the starch solution in the 150 L reactor. The mixture was continuously stirred for 60 min. Then, 37.5 L of AA was gently transferred into the reactor and the mixture was constantly stirred for another 60 min. The starch–AA mixture was then transferred into zip lock plastic bags and subsequently irradiated by gamma radiation at 14 kGy using a ^60^Co gamma irradiator (JS8900-IR155, Nordian, Ottawa, ON, Canada) installed at the Thai Irradiation Center, Thailand Institute of Nuclear Technology (TINT), located in Technopolis, Pathum Thani, Thailand. After irradiation, the mixture turned into gel. The gel was cut into smaller pieces and dried in air oven at 60 °C to yield dried SWA. The total weight of SWA prepared from this up-scaling production was 50 kg. The yield percentage as calculated from the ratio between the total weight of dried SWA (50 kg) to that of the starting materials (51 kg) was 98%.

Degree of grafting (D_g_) was calculated from the weight changes after the grafting process using Equation (1):

D_g_ (%) = (W_2_ − W_1_) × 100/W_1_ (1)

where W_1_ is the weight of cassava starch and W_2_ is the weight of starch-g-PAA (after extraction of homopolymer).

Swelling ratio was gravimetrically determined. Approximately 0.1 g of dried SWA was left to swell in 500 mL of distilled water at ambient temperature. The swollen SWA was removed from the water, at specific time intervals. After the removal of excess water from the surface, the swollen sample was weighed and put back into the water to continue the swelling process. Weight measurements continued for 7 days. The swelling ratio was calculated from Equation (2):

Swelling ratio (g/g) = (W_s_ − W_d_)/W_d_ (2)

where W_d_ is the weight of initial dried SWA and W_s_ is the weight of swollen SWA. Five samples were tested and the results were reported in the form of mean values and their standard deviation.

### 2.3. Available Water Capacity (AWC)

SWA samples were sent to the Department of Soil Science, Faculty of Agriculture, Kasetsart University, Kamphaeng Saen Campus, Kamphaeng Saen District, Nakhon Pathom Province, for analysis of available water capacity (AWC). In addition to our cassava starch-based SWA sample, a cellulose-based SWA prepared in our laboratory and a commercial SWA were also sent for comparative analysis. First, field capacity (FC) and permanent wilting point (PWP) were determined using the pressure plate extractor [60,61,62]. AWC was then calculated from Equation (3).

AWC (%) = FC (%) − PWP (%)(3)

### 2.4. Characterization of SWA

Fourier transform infrared (FTIR) spectra of starch, PAA and SWA (starch-*g*-PAA) were recorded on a Bruker (Tensor 27) FTIR spectrometer. All spectra (16 scans at 4 cm^−1^ resolution) were recorded using attenuated total reflectance (ATR) accessory. Thermal decomposition was analyzed using a Mettler Toledo thermogravimetric analysis (TGA) instrument (TGA/DSC2/LF/1100) at a heating rate of 20 °C/min under an inert (N_2_) atmosphere. Tescan (Vega 3, Brno, Czech Republic) scanning electron microscope (SEM) was used for visualization of morphological structures of dried and swollen SWA. Before SEM experiments, gold coating was applied to all samples.

### 2.5. Field Tests with Young Rubber Trees Planted in Arid Area

The field tests of SWA with young rubber trees were carried out at Chachoengsao Rubber Regulatory Center, Sanam Chai Khet District, Chachoengsao Province. Located in the central region of Thailand and roughly 90 km to the east of Bangkok, Chachoengsao has a tropical climate with an average temperature of 28 °C. December is the driest month and September is the wettest month.

The experiment was implemented based on a randomized complete block design (RCBD), as shown in Figure S1 (available in the supplementary information section), with six different treatments according to the dried weight of SWA, i.e., 0, 10, 20, 30, 40 and 50 g per one rubber tree (T1, T2, T3, T4, T5 and T6, respectively). The RCBD was designed for five replications for each treatment and 12 trees for each replication. The design resulted in a total of 60 trees for each treatment and a total of 360 trees for the entire experiment for one season.

**Figure 1:**
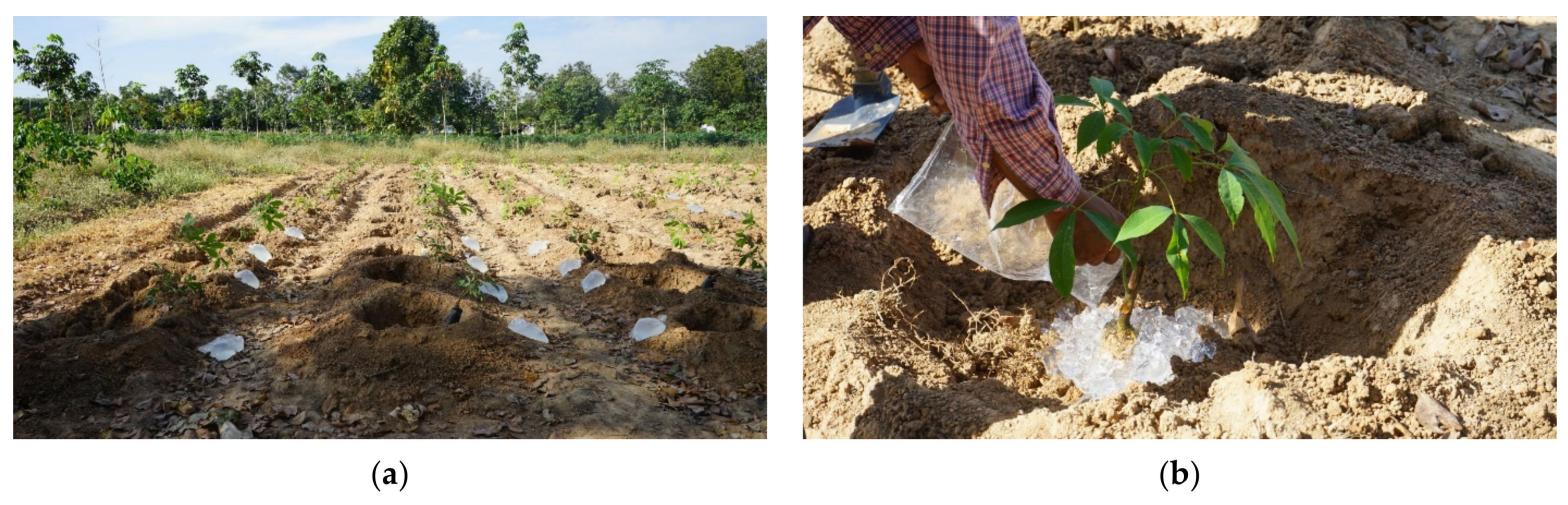
(**a**) Experimental field; (**b**) swollen super water absorbent (SWA) placed under and around the roots of young rubber trees.

Photographs in Figure 1 were taken on 20 December 2016 (designated as Day 1 of season 1), during the plantation of young rubber trees in the experimental field. Dried SWA granules were weighed for each treatment (10, 20, 30, 40 and 50 g) and swollen in local tap water in excess amount (3, 5, 7, 9 and 11 L, respectively) in plastic containers (60 containers for each treatment) for 2 days prior to the planting. On Day 1, 360 young rubber plants were relocated from an adjacent nursery station to the experimental field. A total of 300 bags of swollen SWA were brought in and positioned at their assigned location (T2, T3, T4, T5 and T6).

For T1 treatment, the young rubber trees were planted in a regular approach. The planting materials were placed in the holes. The PE bags were cut and removed before placing the young rubber trees into the holes. For T2–T6 treatments, in addition to the planting materials, the swollen SWAs were put under as well as around the roots of young rubber trees before filling the holes back with soil. The tests were performed twice (season 1 and season 2) in two adjacent fields during different months and years in order to account for environmental variation. For the first season, the experiment started on 20 December 2016 and the data collection continued for the next 6 months until 19 June 2017. For the second season, the experiment began on 13 March 2018 and the data collection was carried on for the next 7 months until 12 October 2018. From the start to the end of the field tests, all young rubber trees were left without watering, except for natural rain, in order to investigate the effects of SWA on survival rate and growth characteristics of young rubber trees planted in arid area. During the experiments, weed control was performed regularly to prevent underdeveloped growth of the young rubber trees. Data collection included the rubber trees’ height, rate of survival and growth characteristics of the young rubber trees.

### 2.6. Statistical Analysis

The statistical analysis was done by data collection from all 60 trees for each treatment, i.e., from all 360 trees for the entire experiment. To compare the survival rate of rubber trees from different treatments, the data were subjected to the analysis of variance (ANOVA) followed by Tukey HSD post hoc test at 0.05 significant level. The data were tested for the validity of normal distribution and homogeneity of variances. In order to study the growth characteristics among the treatments, the number of trees in each characteristic was counted and analyzed using Chi-square test. All tests were performed using Minitab program.

## 3. Results

### 3.1. Degree of Grafting and Swelling Ratio

**Figure 2:**
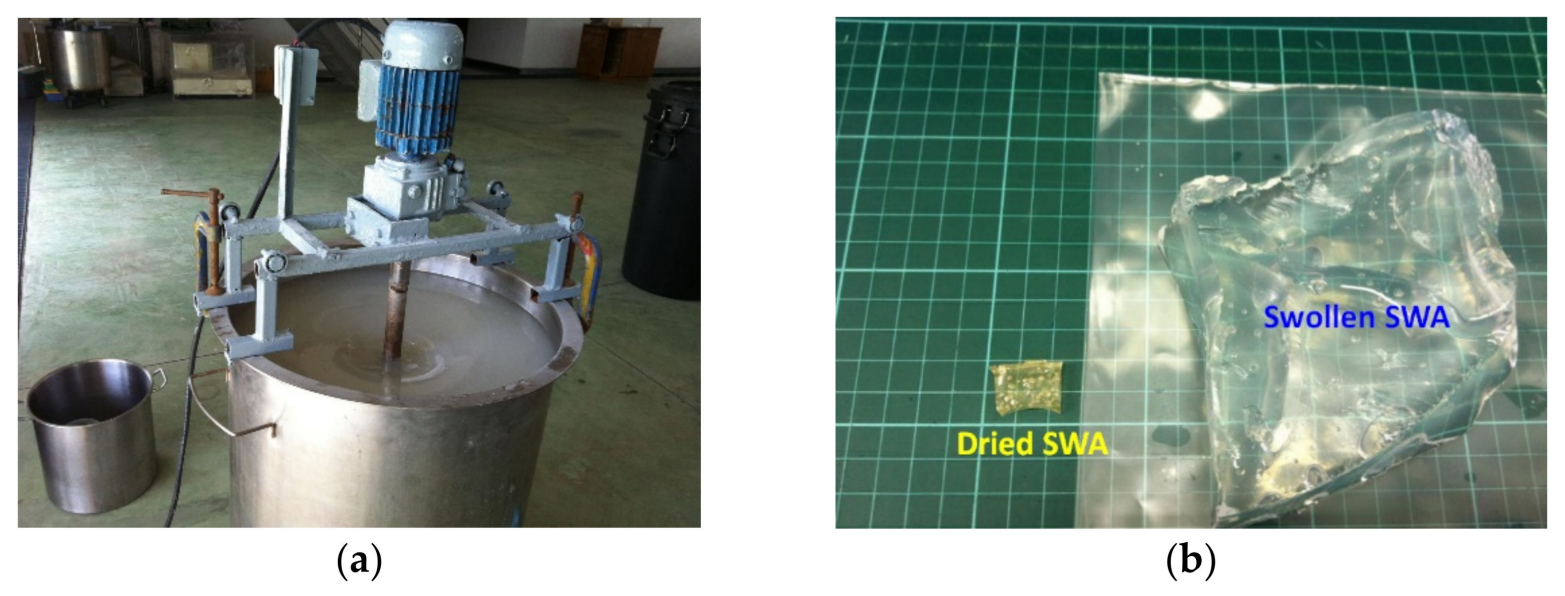
(**a**) A 150-L reactor used for up-scaling production of SWA; (**b**) Physical appearances of dried and swollen SWA.

For the synthesis of SWA in laboratory scale in our previous work [50], starch was gelatinized first by heating the aqueous solution of cassava starch at 80 °C for 60 min. However, the thermal gelatinization of starch was not practical for the up-scaling production due to the difficulties associated with heating large-scale reactor. Hence, gelatinization at room temperature by KOH solution was used for this study. With the removal of the heating process, the up-scaling production of SWA became simplified and more practical and, most importantly, less energy-consuming. Figure 2 shows the large-scale reactor used for the up-scaling production of SWA along with photographs of the synthesized SWA in both dried and swollen forms.

D_g_ and swelling ratio of SWA prepared from the up-scaling production are shown in Table 1, along with those of SWA prepared from the lab-scale synthesis at an optimized condition from our previous work.

**Figure 3:**
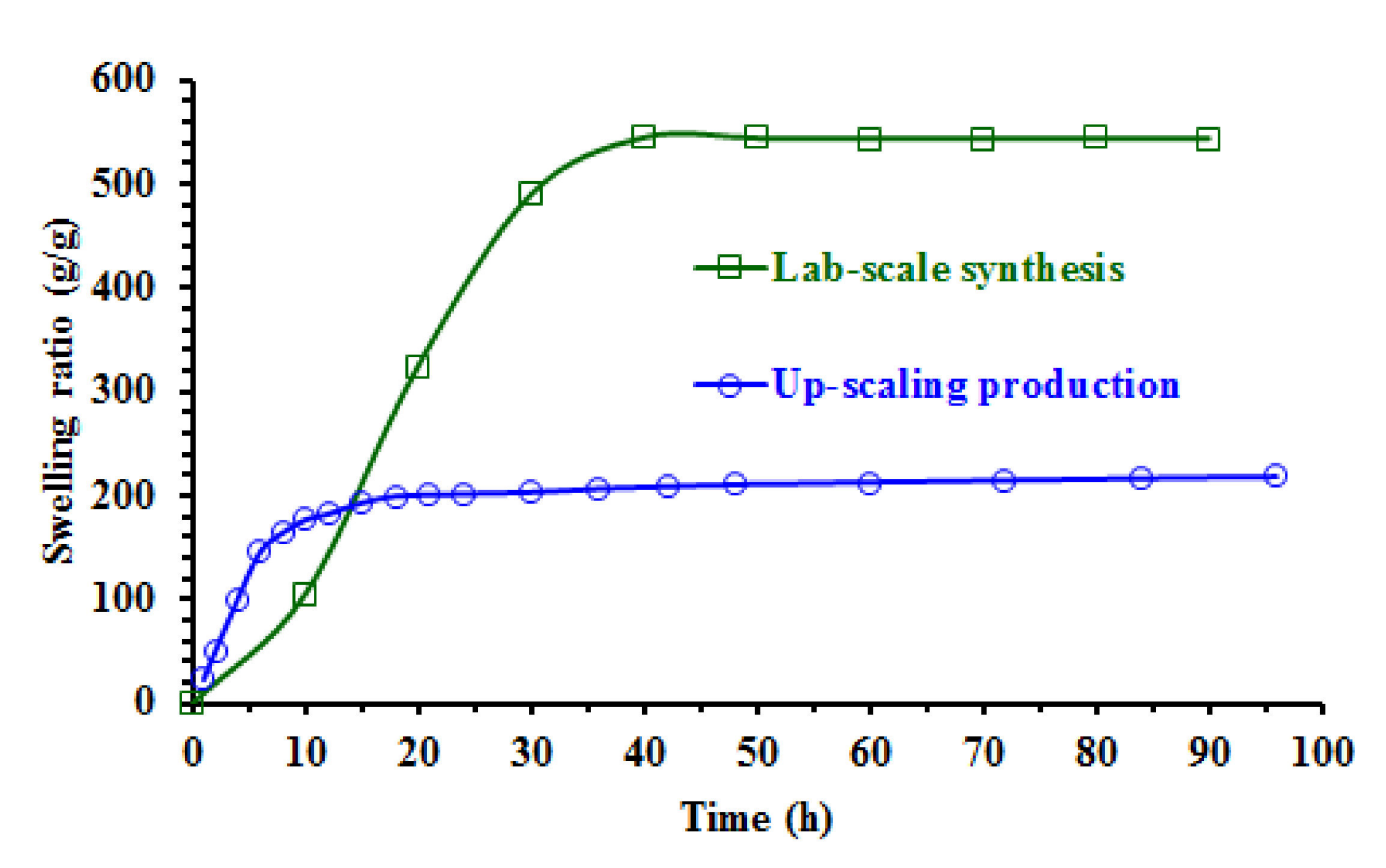
Swelling kinetics of SWA prepared from lab-scale synthesis and up-scaling production.

Figure 3 shows the swelling kinetic of SWA prepared from the up-scaling production and the lab-scale synthesis.

For SWA prepared from the up-scaling production, initially, the swelling rapidly increased with time and seemed to stabilize after 18 h. The swelling ratios after 18, 24, 72 and 96 h were 199.53 (±12.27), 201.56 (±11.38), 214.83 (±10.68) and 218.27 (±10.64) g/g, respectively. For SWA prepared from the lab-scale synthesis, its swelling kinetic seemed to be slower at the very beginning. However, after 15 h, SWA prepared from the lab-scale synthesis was able to absorb water at a much higher rate than SWA prepared from the up-scaling production. The major reasons responsible for the differences in terms of Dg, swelling ratio and swelling kinetic of SWA prepared from these two methods are most likely due to (1) change of thermal gelatinization to alkaline gelatinization (2) change of dose rate and irradiation dose due to a change from lab-scale gamma irradiator to commercial-scale gamma irradiator and (3) change of drying conditions and equipment from small amount of SWA samples prepared from lab-scale synthesis to large amount of SWA samples obtained from up-scaling production. SWA prepared from the up-scaling production resulted in lower swelling ratio than SWA prepared from the lab-scale synthesis. Nevertheless, SWA prepared from the up-scaling production via a green technology of radiation-induced graft polymerization was able to offer a swelling ratio over 100 g/g which was more than enough to offer a great potential as a promising and sustainable product for agricultural applications. More importantly, the change from the thermal gelatinization in the lab-scale synthesis to the alkaline gelatinization in the up-scaling production made the process more simplified and practical as well as less energy-consuming, since it requires no heating for the starch gelatinization.

### 3.2. Available Water Capacity (AWC)

Generally, plants cannot access all existing water in soil. AWC is the difference between field capacity (the maximum amount of water that soil can hold) and wilting point (the point at which plants can no longer extract water from soil). Beyond the wilting point, the remaining water kept in pores that are too small for plant roots to retrieve is considered unavailable water. The available water in soil is one of the key factors that are vital for plant developments, since it provides dissolved organic and inorganic substances containing essential elements and crucial nutrients that are accessible for plant uptake via absorption through plant roots.

The ability of SWA prepared in this study to function as a water reservoir was quantitatively tested using AWC analysis. The results are shown in Table 2, in comparison with another cellulose-based SWA prepared in our laboratory and another commercially available SWA. From Table 1, among the three samples, the cassava starch-based SWA has the highest value of AWC at 5511%, which is much higher than 2987% of the commercial SWA. The cellulose-based SWA shows the lowest value at 1278%, this is probably due to the fact that the cellulose used for the preparation of SWA was used as-is and not delignified. Without delignification, the grafting efficiency was comparatively low, thus resulting in SWA with lower water absorption. The results from Table 2 indicated that the cassava starch-based SWA prepared by radiation-induced graft polymerization was able to store much more amount of available water, compared with the commercial SWA and the un-delignified cellulose-based SWA.

### 3.3. Characterization of SWA

**Figure 4:**
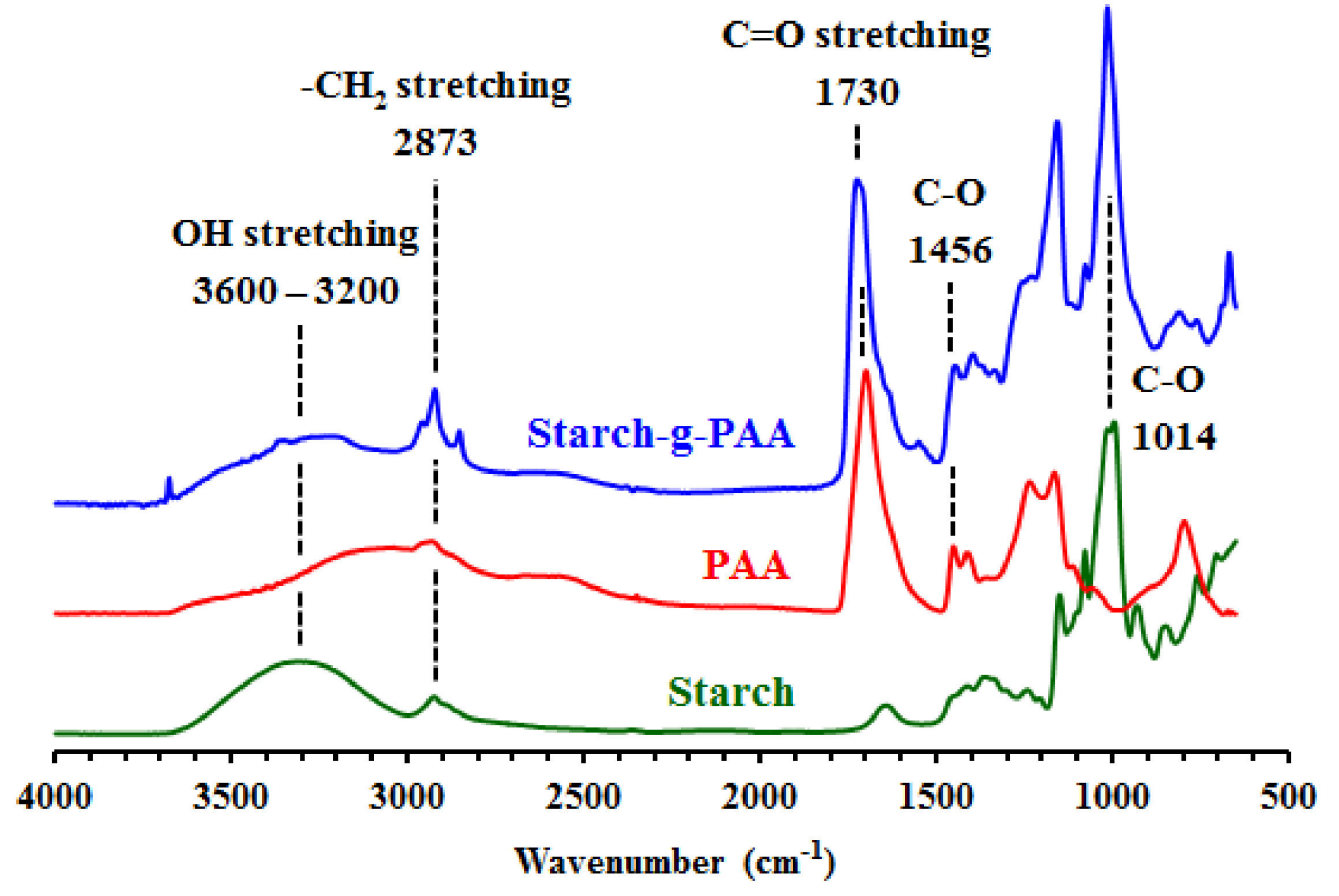
FTIR spectra of starch, polyacrylic-acid-(PAA) and starch-*g*-PAA (SWA).

After the synthesis, the chemical, thermal and morphological characterization of the obtained SWA was performed by FTIR, TGA and SEM, respectively. Figure 4 shows the results from the characterization of starch, PAA and SWA (starch-*g*-PAA) by FTIR.

In Figure 4, the stretching vibrations of O–H, C–H and C–O bonds, existing in the chemical structure of starch, were observed at 3600–3200, 2873 and 1014 cm^−1^, respectively [63]. For PAA, the stretching vibration of C=O and C–O bonds, present in –COOH functional groups, can be seen at 1730 and 1456 cm^−1^, respectively. FTIR spectrum of SWA showed vibration peaks at 3600–3200, 2873, 1730, 1465 and 1014 cm^−1^. The existence of these aforementioned peaks of O–H, C–H and C–O bonds observed in the FTIR spectrum of starch and those of C=O and C–O bonds in the FTIR spectrum of PAA, in a single FTIR spectrum of SWA, was a solid confirmation that graft polymerization of AA onto starch could be induced and accomplished by gamma radiation.

**Figure 5:**
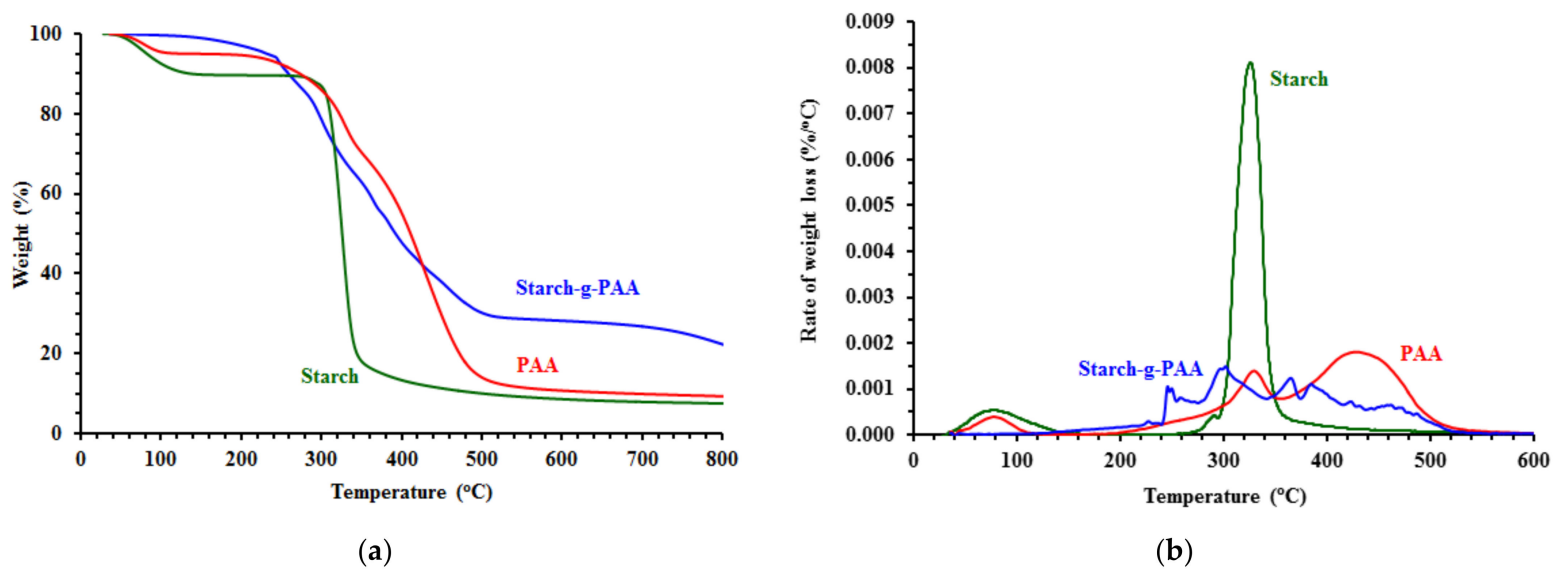
(**a**) TGA thermograms of starch, PAA and SWA; (**b**) dTGA thermograms of starch, PAA and SWA.

Figure 5a shows the TGA thermograms of CS, PAA and SWA, while Figure 5b displays their corresponding dTGA thermograms.

Figure 5a,b show that thermal degradation of starch occurred in two major steps. Centering at around 80 °C, the first step was due to vaporization of moisture. Attributable to the major thermal degradation of polymeric backbone of starch, the second step reached its maximum rate of weight loss at around 320 °C and eventually resulted in 7.5% of char yield at 800 °C. From Figure 5a,b, PAA thermally degraded in three main processes. The first two processes were similar to those of starch. However, the major process for PAA was the third one, taking place between 360–540 °C and leading to 9.3% of char yield at 800 °C. Unlike starch and PAA, SWA thermally decomposed without any major degradation process before 240 °C. After 240 °C, a multi-stage weight loss process took place, showing a number of peaks with high rate of weight loss at around 250, 300, 370 and 380 °C. The degradation continued until 540 °C. At 800 °C, the char yield of SWA was roughly 22%. The successful synthesis of SWA from radiation-induced graft polymerization of AA onto starch was thus confirmed by the distinctive thermal degradation pattern and char yield of SWA.

**Figure 6:**
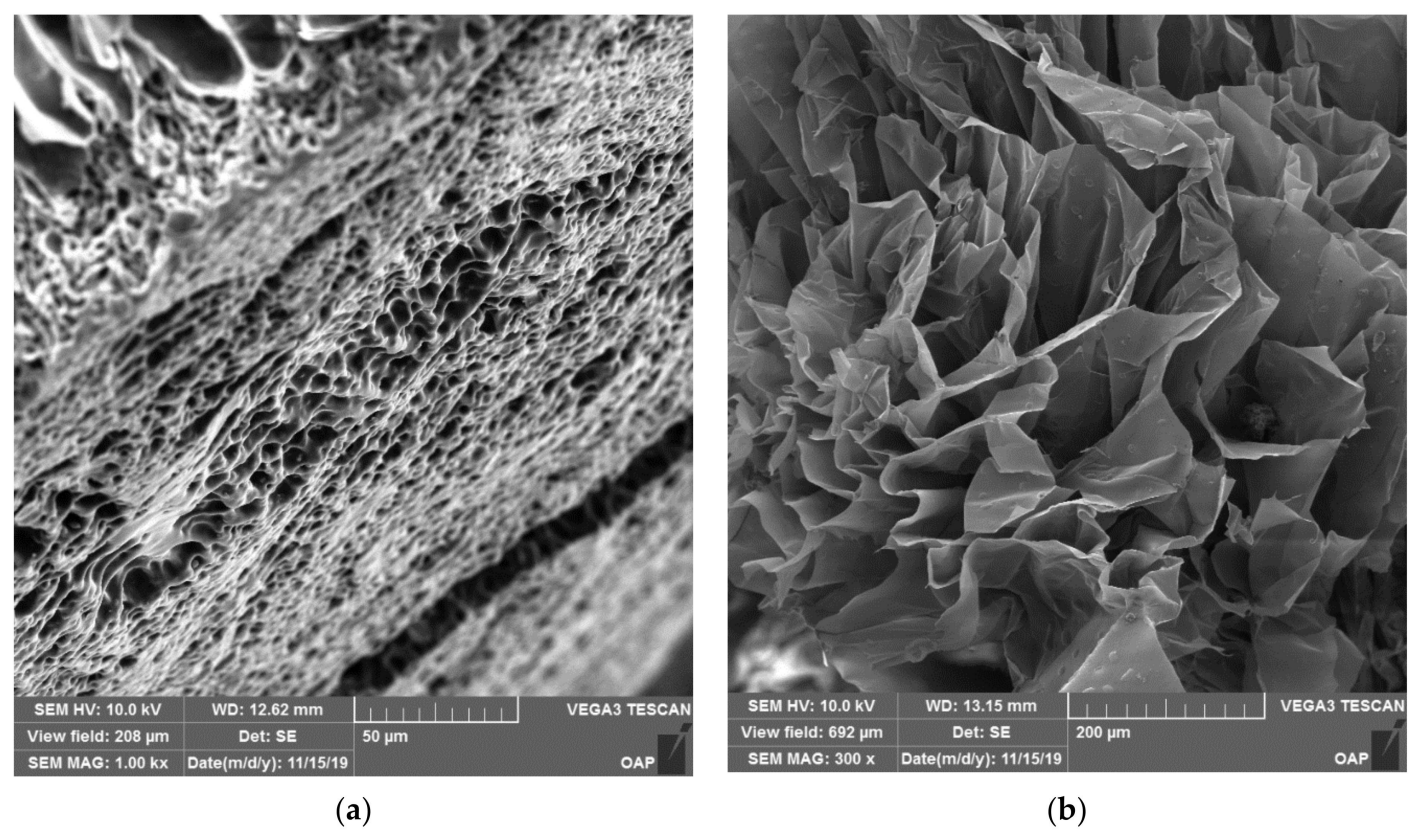
(**a**) SEM micrograph at magnification 1000× of synthesized SWA after irradiation; (**b**) SEM micrograph at magnification 300× of swollen SWA.

The morphological structures of SWA samples analyzed by SEM are shown in Figure 6. Figure 6a shows SEM image at magnification 1000× of the SWA sample obtained after irradiation. The prepared SWA exhibits a highly coarse surface with countless microscopic pores. Generally, the unmodified, original cassava starch has a relatively smooth surface [64]. The loss in smoothness of SWA was due to the graft polymerization of PAA onto starch surface. SEM image at magnification 300× of the fully swollen SWA is displayed in Figure 6b. After irradiation, the prepared SWA was washed with water and dried to a constant weight in an air oven. The dried SWA was left in water for 48 h to have the sample saturated with water. The swollen SWA sample was freeze dried and subsequently cross-sectional cut prior to SEM analysis. The SEM micrograph of the swollen SWA clearly displayed highly porous network structure with plentiful spacious pores. The loose porous structure clearly explained the observed high swelling ability of SWA. The interconnected honeycomb-like large pores offered passages for water permeation through the network [22]. From these results, high water absorbency and great swelling ability were apparently attributable to the presence of the hydrophilic groups in SWA’s chemical structure together with its highly porous morphology.

### 3.4. Field Tests with Young Rubber Trees Planted in Arid Area

**Figure 7:**
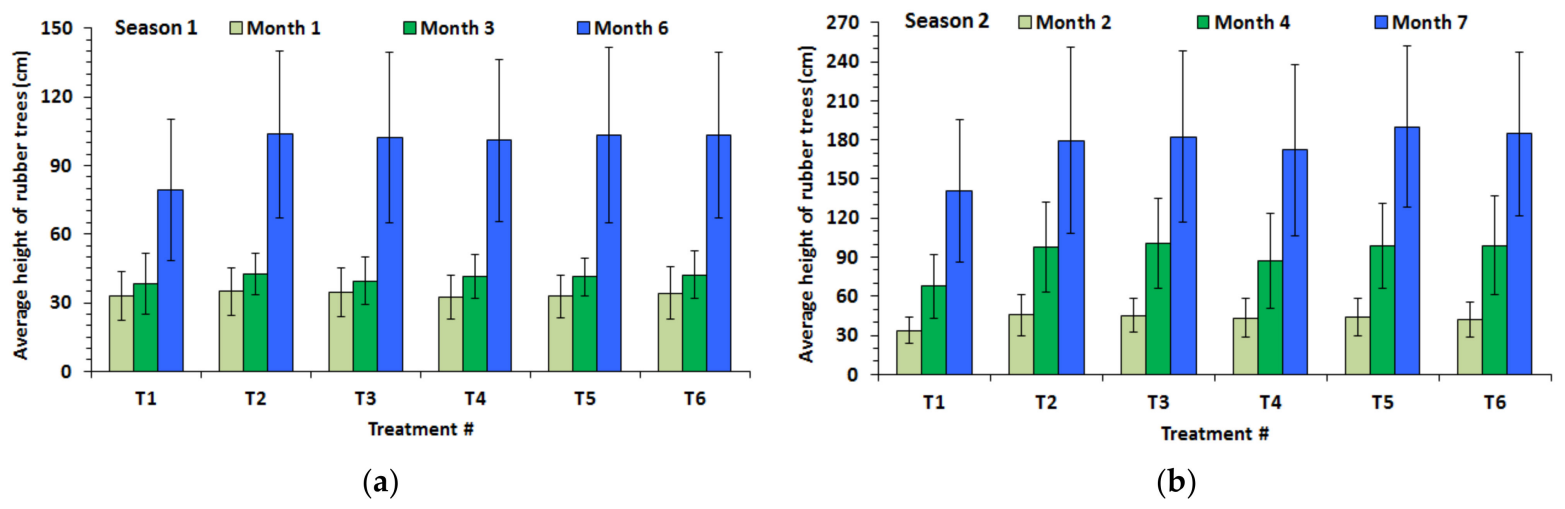
(**a**) Effects of SWA on average height of rubber trees from the experiment performed during season 1 after 1, 3, and 6 months, without watering; (**b**) Effects of SWA on average height of rubber trees from the experiment performed during season 2 after 2, 4 and 7 months, without watering.

Figure 7 shows mean values for the rubber trees’ height for each treatment from the experiment performed during season 1 and season 2. For season 1, after 1 month, the average heights of all six treatments were fairly similar, varying between 32 and 35 cm. After 3 months, the mean heights remained comparatively similar, between 38 and 43 cm. After 6 months, the average height of the rubber trees from the control treatment was about 80 cm, whereas those from the SWA treatments were in 100–104 cm range.

For season 2, after 2, 4 and 7 months, the averaged values for the rubber trees’ height of the control treatment were 34, 68 and 141 cm, in that order, while those from the SWA treatments were in the range of 42–45, 98–100 and 180–190 cm, respectively. Comparatively, the average height of rubber trees from season 2 seemed to be higher than those from season 1. One of the major reasons for this difference is most likely the diverse rainfall amount during the experiments performed in season 1 and season 2. The 30-year averaged values for rainfall amount and rainy days in Chachoengsao Province along with the values collected during the experiments from season 1 and season 2 are summarized in Table S1, (available in the supplementary information section) [65]. From Table S1, it is obvious that both the collective rainfall amount (883 mm) and the rainy days (61 days) collected during 6 months in season 1 were much lower than those collected during 7 months in season 2.

**Figure 8:**
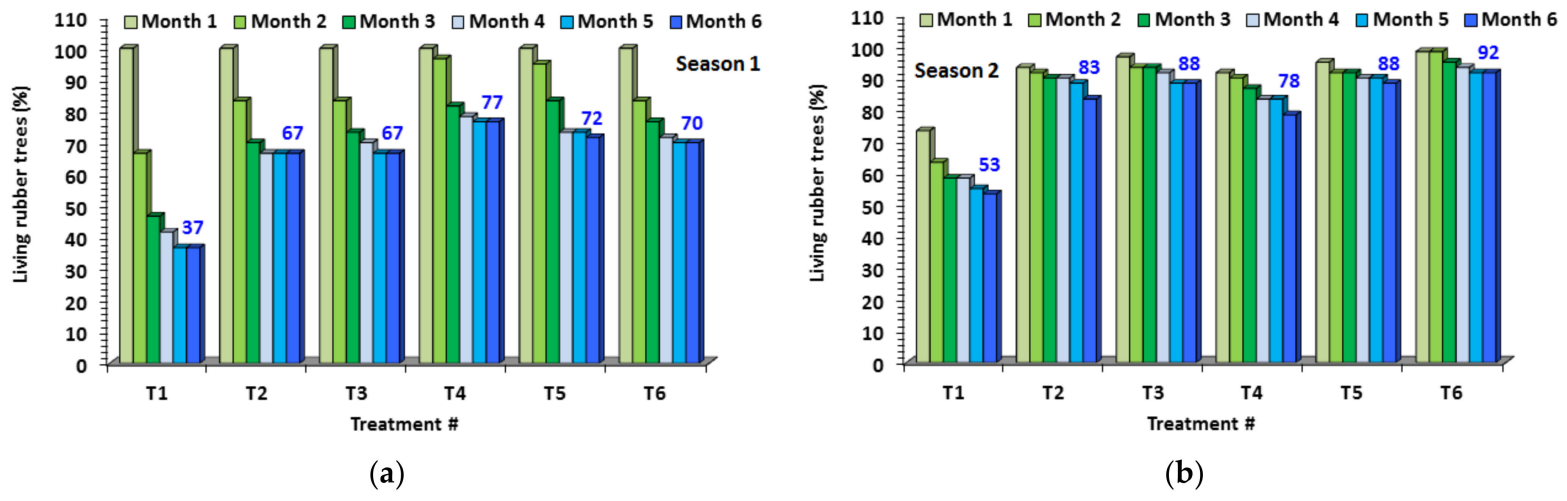
(**a**) Effects of SWA on percentage of living rubber trees from the experiment performed during season 1 after 6 months, without watering; (**b**) effects of SWA on percentage of living rubber trees from the experiment performed during season 2 after 6 months, without watering.

Data collection for the field test with rubber trees for the rate of survival was monthly done for 6 months after plantation. Figure 8 shows the monthly percentage of living rubber trees for each treatment. For season 1, the living rubber trees from the control treatment (T1) drastically dropped from 100% (after one month) to 67% and 47% after two and three months, respectively. The lack of water was the main reason behind the lower percentage of living rubber trees for the control treatment. After that, the percentage of living rubber trees continued to fall, at a much slower rate. This was consistent with local knowledge from rubber farmers, that if the young rubber trees could survive the first 3 months after plantation, they had a much higher chance to survive after that. At the end of the experiment, after 6 months, the number of living rubber trees for the control treatment was 22 (or 37%). For the SWA treatments, the rate for the decline of the percentage of living rubber trees was not as drastic as that of the control treatment, dropping from 100% after one month to 83–97% and 70–83% after two and 3 months, respectively. After 3 months, the drop of the percentage of living rubber trees stabilized or even remained unchanged. After 6 months, the percentage of living rubber trees for the SWA treatments was between 67–77%, which was 30–40% higher than that of the control treatment.

For season 2, after 1 month, the living percentage of the young rubber trees was 73, 93, 97, 92, 95 and 98, for T1, T2, T3, T4, T5 and T6, respectively, which was already lower than 100%. A plausible explanation for this is the fact that the plantation was done on 13 March 2018 which was the beginning of summer season in Thailand. The first month of season 2 was between 13 March and 12 April 2018. This period is generally the hottest time of the year, when temperature can reach over 40 °C. For the following months, season 2 showed gradual decline of the percentage of living rubber trees. After 6 months, the percentage of living rubber trees for the control treatment was 53, while that of SWA treatment was 83, 88, 78, 88 and 92 for T2, T3, T4, T5 and T6, respectively. The percentage of living rubber trees after six months from season 2 was higher than that of the same treatment from season 1. Once again, this is most likely due to the obvious difference in terms of the amount of rainfall and rainy days collected during the experiments. For season 2, the results implied that the presence of SWA resulted in 25–39% increase in survival rate of young rubber trees. Results from the statistical analysis through Tukey method revealed that, for both season 1 and season 2, the average survival rate of T1 was statistically significant and different from that of T2, T3, T4, T5 and T6 (*p* < 0.05), whereas there was no statistically significant difference among the average survival rate of T2, T3, T4, T5 and T6 (*p* > 0.05).

For season 1, the highest rate of survival for the young rubber trees was 77%, which was achieved by using 30 g of SWA per one rubber tree (T4). For season 2, the application of 50 g of SWA per one rubber tree (T6) resulted in the maximum rate of survival (92%) of the young rubber trees. However, as mentioned earlier, the major differences between season 1 and season 2 were the amount of rainfall and the number of rainy days, with season 1 having a lot less rainfall and rainy days than season 2. As a result, the optimum amount of SWA suitable for increasing the survival rate of the young rubber trees planted in arid areas in dry season was determined to be 30 g of SWA per one tree.

## 4. Discussion

The considerable increase in survival rate of rubber trees grown in the presence of SWA can make an impact in productivity, for the rubber farmers in arid areas, enabling them to save cost and time. In addition to labor costs for watering and irrigation, SWA can help rubber farmers save cost from money lost due to the loss of rubber trees as well as money to be spent for purchasing new young rubber trees to replace for the dead ones. Additionally, the utilization of fully swollen SWA makes it possible for the retained water to be utilized to its utmost efficacy, since available water can be kept in the swollen SWA and will not be wasted through drainage via direct watering. This is an ideal situation for drought affected areas where water scarcity has become a critical concern.

**Figure 9:**
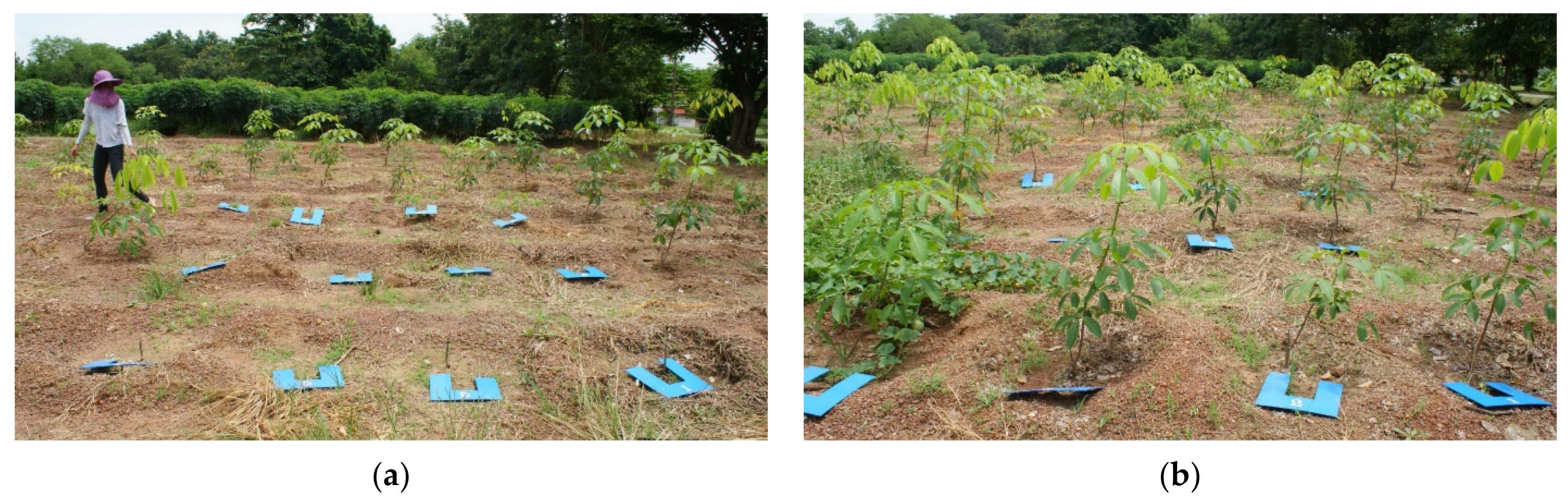
(**a**) A picture of rubber trees taken from one block of control treatment after 6 months, without watering; (**b**) a picture of rubber trees taken from one block of SWA treatment after 6 months, without watering.

Figure 9 shows photographs of the remaining living rubber trees taken after 6 months from one block from the control treatment and one block from the SWA treatment, from season 1. Without SWA, the majority of the rubber trees were dead, whereas the surviving ones were relatively small. On the contrary, with SWA, most of the rubber trees thrived, were healthy and comparatively tall. In order to differentiate the wellness and strength of the remaining living rubber trees from each treatment, the data on the growth characteristics of the rubber trees were also gathered, monthly, as shown in Figures S2–S4 (available in the supplementary information section).

The results of this study proved that SWA offers promising potential for agricultural applications. The results are consistent with those previously reported in similar studies. Demitri et al. [53] thoroughly examined the potential of cellulose-based superabsorbent hydrogels as water reservoirs in agriculture. The study enumerated a number of advantages of superabsorbent hydrogels. The following are reasons for some of the benefits of SWA on plants’ growth, productivity, survival rate and growth characteristics. When mixed with soil in areas adjacent to the root zone of a plant, dried SWA can absorb water from irrigation or rain, thus reducing the loss of water from evaporation and drainage. Once saturated with water, swollen SWA can act as a local reservoir, effectively maintaining moisture balance, directly and gradually releasing small amounts of retained water (to the plant roots as the soil dries) and thus steadily keeping the soil moist for weeks or even months.

Besides being a local water reservoir for soil, the swollen SWA offers additional marvelous values for the soil and the plant’s roots. Sannio et al. [7] described the swelling effects of the hydrogels on the soil, elucidating the fact that superabsorbent hydrogels can lead to increased soil porosity and consequently better oxygenation. This explanation is in good agreement with our results from a simple experiment to follow the change of a fully swollen SWA sample (kept under sandy soil) with time, as shown in Figure S5, (available in the supplementary information section). With more oxygen available for the plants’ roots, the improved soil aeration and better oxygenation are most likely one of the major reasons for better growth, productivity and growth characteristics of plants grown in the presence of SWA.

## 5. Conclusions

Cassava starch-based SWA was successfully synthesized in an up-scaling production by radiation-induced graft polymerization of AA onto CS. The change from the thermal gelatinization in the lab-scale synthesis to the alkaline gelatinization in the up-scaling production made the process more simplified and practical as well as less energy-consuming. For the first time, two seasons of a field test of starch-based SWA with young rubber trees were carried out in a comparatively sizable scale, with a large number of samples. The field tests with young rubber trees planted in an arid area without any further watering except natural rain demonstrated that SWA was capable of increasing the survival rate of the young rubber trees, while concurrently enhancing their growth characteristics. The optimized amount of SWA required to maximize the survival rate of the young rubber trees planted in arid areas in dry season was 30 g of SWA per one rubber tree. The ability of SWA to initially retain a large amount of water and subsequently and gradually release water to the root zone of the plants can result in a higher survival rate of plants grown in arid areas, while the increased soil porosity and better oxygenation can bring about enhanced growth characteristics. The benefits therefore include reducing water consumption, decreasing frequency of watering, increasing soil porosity and enabling better oxygenation. Consequently, SWA offers a promising and sustainable way of increasing survival rate of trees planted in arid areas.

## Figures and Tables

**Table 1:** D_g_ and swelling ratio of SWA prepared from the lab-scale synthesis (at an optimized condition) and the up-scaling production.

Preparation Method	D_g_ (%)	Swelling Ratio (g/g)
Lab-scale synthesis	260.48	543.93
Up-scaling production	594.68	218.27

**Table 2:** Available water capacity (AWC) of three different SWA samples.

SWA Samples	Available Water Capacity (AWC) (%)
Cassava starch-based	5511
Cellulose-based	1278
Commercial	2987

## Data Availability

The data presented in this study are available upon request.

## Supplementary Materials

The following are available online at https://www.mdpi.com/article/10.3390/polym13081314/s1, Figure S1: Location for the experimental field and the RCBD design for SWA’s field test with young rubber trees, using six different treatments, with five replications for each treatment and 12 trees for each replication, Figure S2: Ten different growth characteristics of the young rubber trees, Figure S3: Effects of SWA on monthly growth characteristics of all 60 young rubber trees from each treatment, from the experiment performed during season 1 for six months, without watering, Figure S4: Effects of SWA on monthly growth characteristics of all 60 young rubber trees from each treatment, from the experiment performed during season 2 for six months, without watering, Figure S5: Change of swollen SWA sample and its surrounding sandy soil with time, Table S1: 30-year averaged values for rainfall amount and rainy day in Chachoengsao Province and the values collected during the experiments from season 1 and season 2.
