# Supplementary material for: Starch-Based Super Water Absorbent: A Promising and Sustainable Way to Increase Survival Rate of Trees Planted in Arid Areas
Source: Polymers (Basel). 2021 Apr 16;13(8):1314. doi: 10.3390/polym13081314 (PMC8073703; doi:10.3390/polym13081314)
Supplement: Supplementary file 1 [file polymers-13-01314-s001.zip › polymers-1138925-supplementary.pdf]

polymers

MDPI

Article

Starch-based Super Water Absorbent: A Promising and Sustainable Way to Increase Survival Rate of Trees Planted in Arid Areas

Pattra Lertsarawut [1], Thitirat Rattanawongwiboon [1], Theeranan Tangthong [1], Sakchai Laksee [1], Tanagorn Kwamman [1], Butri Phuttharak [2], Phayao Romruensukharom [2], Phiriyatorn Suwanmala [1] and Kasinee Hemvichian [1,*]

[1] Thailand Institute of Nuclear Technology (Public Organization), Nakhon Nayok 26120 Thailand; pattra@tint.or.th (P.L.); thitirat@tint.or.th (T.R.); t_ranan@hotmail.com (T.T.); sakchai@tint.or.th (S.L.); tanagorn@tint.or.th (T.K.); piriyatorn@tint.or.th (P.S.)
[2] Chachoengsao Rubber Regulatory Center, Chachoengsao 24160 Thailand; bp_shan@hotmail.com (B.P.); phayaor@hotmail.com (P.R.)
* Correspondence: kasineeh@yahoo.com

Citation: Lertsarawut, P.; Rattanawongwiboon, T.; Tangthong, T.; Laksee, S.; Kwamman, T.; Phuttarak, B.; Romruensukharom, P.; Suwanmala, P.; Hemvichian, K. Super Water Absorbent: A Promising and Sustainable Way to Increase Survival Rate of Trees Planted in Arid Areas. *Polymers* **2021**, *13*, 1314. https://doi.org/10.3390/polym13081314

Academic Editor: Antonio Pizzi, George Z. Papageorgiou

Received: 23 February 2021
Accepted: 14 April 2021
Published: 16 April 2021

Publisher's Note: MDPI stays neutral with regard to jurisdictional claims in published maps and institutional affiliations.

Copyright: © 2021 by the authors. Licensee MDPI, Basel, Switzerland. This article is an open access article distributed under the terms and conditions of the Creative Commons Attribution (CC BY) license (http://creativecommons.org/licenses/by/4.0/).

Supplementary Information

1. RCBD Design

Figure S1 displays an overall picture of the RCBD for the field test, with 5 replications for each treatment and 12 trees for each replication. The design resulted in a total of 60 trees for each treatment and a total of 360 trees for the entire experiment for one season. The field size for the experiment was 800 m² (20 m x 40 m) with spacing between rubber trees at 1 m x 2 m, at a total of 18 rows, with 20 rubber trees in each row. Soil in the experimental field was classified as sandy-clay-loam, with an acidic pH of 5.5 – 5.8.

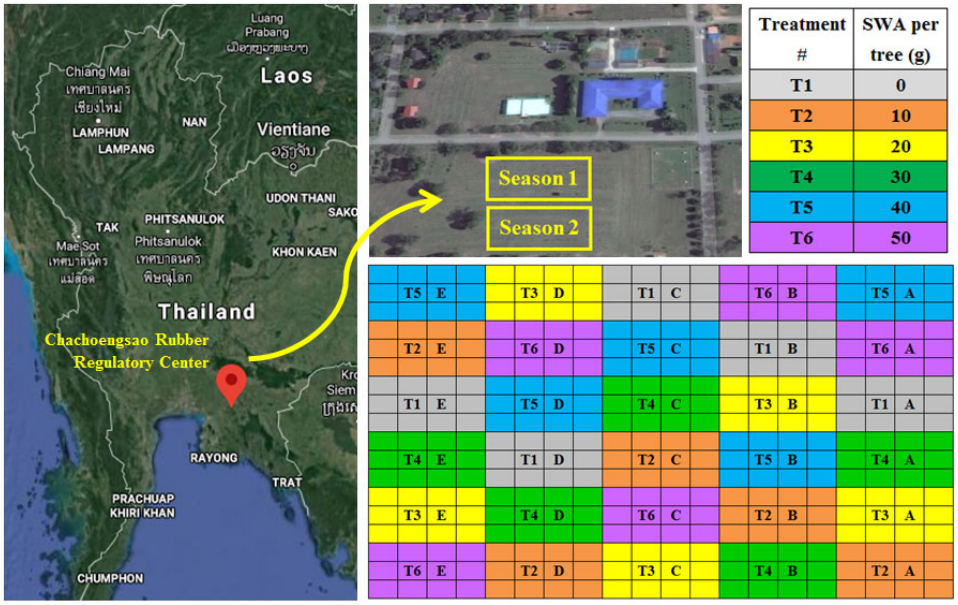

Treatment #	SWA per tree (g)
T1	0
T2	10
T3	20
T4	30
T5	40
T6	50

Figure S1. Location for the experimental field and the RCBD design for SWA's field test with young rubber trees, using 6 different treatments, with 5 replications for each treatment and 12 trees for each replication.

2. Rainfall Amount and Rainy Days in Chachoengsao Province

The 30-year averaged values for rainfall amount and rainy days in Chachoengsao Province [1] along with the values collected during the experiments from season 1 and season 2 are summarized in Table S1. From Table S1, it is obvious that both the collective rainfall amount (883 mm) and the rainy days (61 days) collected during 6 months in season 1 were much lower than those collected during 7 months in season 2. Interestingly, the collective rainfall amount (1,529.4 mm) and the rainy days (117 days) collected during 7 months in season 2 were even higher than the 30-year annual averaged values.

Table S1. 30-year averaged values for rainfall amount and rainy day in Chachoengsao Province and the values collected during the experiments from season 1 and season 2.

Month	30-year averaged values (1961-1990)		Season 1 (from 20 December 2016 to 19 June 2017)		Season 2 (from 13 Mar 2018 to 12 October 2018)	
	Rainfall amount (mm)	Rainy day (days)	Rainfall amount (mm)	Rainy day (days)	Rainfall amount (mm)	Rainy day (days)
January	3.0	1	15.1	4		
February	14.5	2	5.4	3		
March	70.9	11	140.8	10	36.8	2
April	48.3	6	105.1	11	239.4	12
May	147.7	16	413.4	19	342.7	23
June	125.2	12	202.2	13	116.9	16
July	190.5	13			154.4	20
August	170.7	17			127.4	19
September	277.5	17			441.3	18
October	231.9	13			70.5	7
November	22.9	5				
December	0	0	1.0	1		
**Total**	**1303.1**	**113**	**883.0**	**61**	**1529.4**	**117**

3. Growth Characteristics of the Young Rubber Trees

In order to differentiate the wellness and strength of the remaining living rubber trees from each treatment, the data on the growth characteristics of the rubber trees were also gathered, monthly. Figure S2 shows 10 different growth characteristics or the rubber trees classified as (1) dead, (2) no leaves, (3) withered, (4) wilting, (5) normal, (6) budding, (7) 2nd layer, (8) 3rd layer, (9) 4th layer and (10) 5th layer, with "dead" representing the weakest character and "5th layer" symbolizing the strongest character.

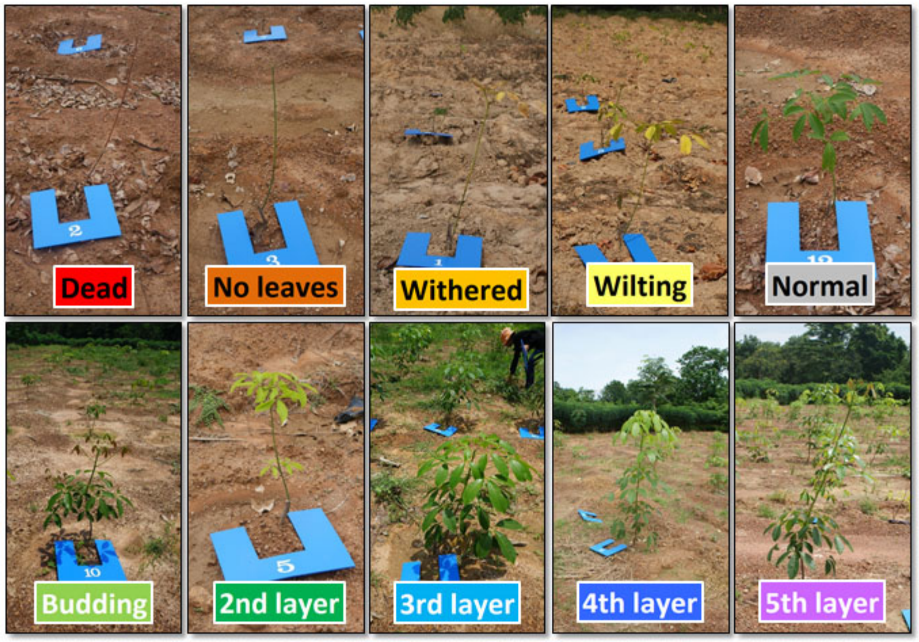

Figure S2. 10 different growth characteristics of the young rubber trees.

The results for the effects of SWA on monthly growth characteristics of the rubber trees are summarized in Figures S3 and S4. Figures S3 and S4 qualitatively as well as quantitatively illustrates both the survival rate and the growth characteristics of the rubber trees from each treatment for each passing month. From Figures S3 and S4, it is obvious that for both season 1 and season 2, from the beginning to the end of the experiment, the young rubber trees from SWA treatments offered better growth characteristics than those from the control treatment. In comparison, for the same treatment at the same time, the young rubber trees from season 2 had better growth characteristics than those from season 1. For both season 1 and season 2, after 6 months, the majority of the living trees from the SWA treatments progressively flourished to the third, fourth and fifth layer with higher percentage than those from the control treatment. Results from the statistical analysis once again endorsed that, for both season 1 and season 2, after 6 months, a significant difference did exist between the growth characteristic of T1 and that of T2, T3, T4, T5 and T6 ($p < 0.05$), while there was no statistically significant difference among the growth characteristic of T2, T3, T4, T5 and T6 ($p > 0.05$).

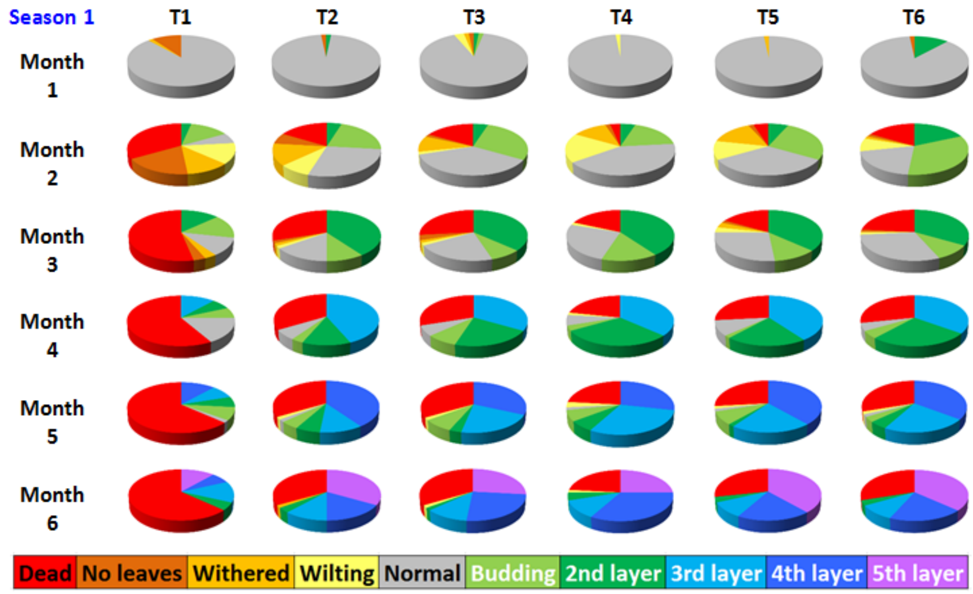

Figure S3. Effects of SWA on monthly growth characteristics of all 60 young rubber trees from each treatment, from the experiment performed during season 1 for 6 months, without watering.

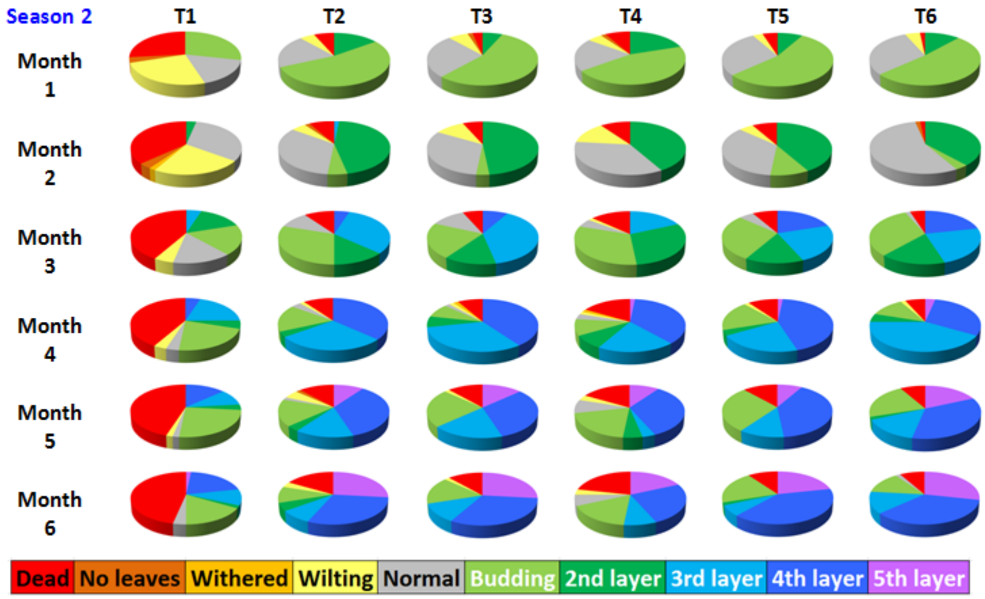

Figure S4. Effects of SWA on monthly growth characteristics of all 60 young rubber trees from each treatment, from the experiment performed during season 2 for 6 months, without watering.

4. Change of a Fully Swollen SWA Sample with Time

Figure S5 shows a simple experiment to follow the change of a fully swollen SWA sample (kept under sandy soil) with time. The fully swollen SWA sample was put under the sandy soil in a clear plastic container. The container was horizontally and vertically marked with blue lines, forming rectangles which are 2 cm wide and 2 cm long. The rectangles were used as an internal standard to follow the dimensional changes of SWA.

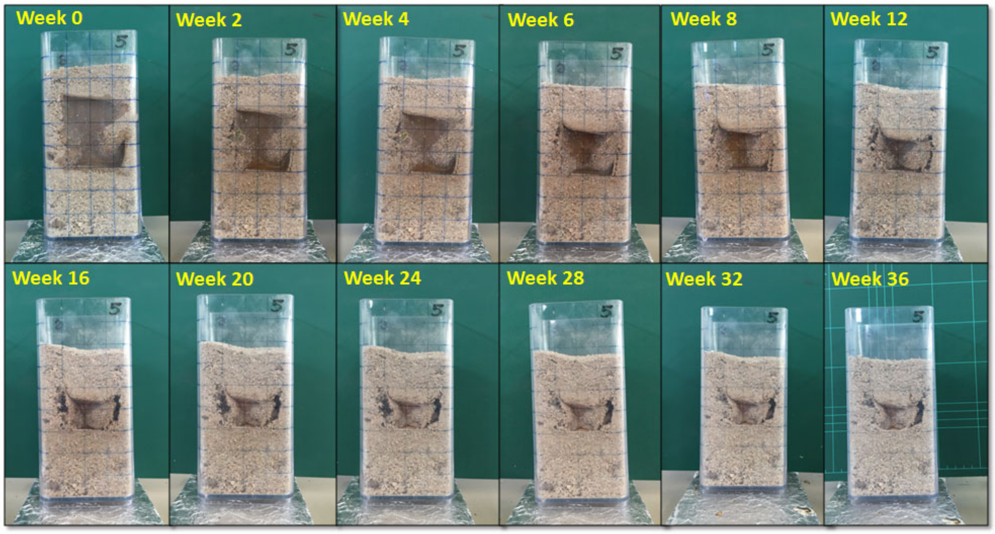

Figure S5. Change of swollen SWA sample and its surrounding sandy soil with time.

At the beginning of the experiment, the swollen SWA made the soil that was in direct contact with its surface humid. After 2 weeks, the surrounding sandy soil was moist, except at the very top and bottom. After 8 weeks (2 months), the soil remained mostly damp, while the swollen SWA was smaller in size (from 6 to 4 occupied rectangles), due to the release of the stored water. As time passed, the SWA sample gradually contracted. With each passing week, the smaller SWA resulted in bigger free space (previously occupied by fully swollen SWA), hence facilitating more air flow and leading to enhanced soil porosity and ultimately better oxygenation.

Author Contributions: Conceptualization, P.S. and K.H.; methodology, B.P., P.R., P.S. and K.H.; validation, P.L., T.R., T.T., S.L. and T.K.; formal analysis, B.P., P.R. and K.H.; investigation, P.L., T.R., T.T., S.L., T.K. and K.H.; resources, B.P., P.R., P.S. and K.H.; writing—original draft preparation, K.H.; writing—review and editing, P.L., T.R., S.L., T.K., P.S. and K.H.; visualization, P.S. and K.H.; supervision, P.S. and K.H.; project administration, P.S. and K.H.; funding acquisition, P.S. and K.H. All authors have read and agreed to the published version of the manuscript.

Funding: This research was funded by the National Research Council of Thailand (NRCT), Ministry of Higher Education, Science, Research and Innovation (MHESI), grant number 2560190602011 and The APC was funded by Thailand Institute of Nuclear Technology (Public Organization).

Acknowledgments: The authors would like to acknowledge the Office of Atoms for Peace (OAP) for their kind support for the scanning electron microscope used for the morphological characterization of SWA samples.

Conflicts of Interest: The authors declare no conflict of interest.

References

1. Thai Meteorological Department, Statistics, Chachoengsao. Average temperature and rainfall during 30-year period (1961-1990). Available online: https://www.tmd.go.th/en/province_weather_stat.php?StationNumber=48458 (accessed 28 January 2021).
